# Cut-off points of the visceral adiposity index (VAI) identifying a visceral adipose dysfunction associated with cardiometabolic risk in a Caucasian Sicilian population

**DOI:** 10.1186/1476-511X-10-183

**Published:** 2011-10-19

**Authors:** Marco C Amato, Carla Giordano, Maria Pitrone, Aldo Galluzzo

**Affiliations:** 1 Dipartimento Biomedico di Medicina interna e specialistica (Dibimis), Section of Endocrinology, University of Palermo; Piazza Cliniche 2, 90127 Palermo, Italy

**Keywords:** Obesity, cardiometabolic risk, visceral adipose dynsfunction, VAI

## Abstract

**Background:**

The Visceral Adiposity Index (VAI) is a sex-specific mathematical index, based on Waist Circumference (WC), Body Mass Index (BMI), triglycerides (TG) and HDL cholesterol (HDL) levels, indirectly expressing visceral adipose function and insulin sensitivity. Our aim was to find the optimal cut-off points of VAI identifying a visceral adipose dysfunction (VAD) associated with cardiometabolic risk in a Caucasian Sicilian population.

**Methods:**

Medical check-up data of 1,764 Primary Care patients (PC patients) were retrospectively and cross-sectionally examined using a receiver-operating characteristic (ROC) curve to determine appropriate stratified-for-age cut-off of VAI, for the identification of PC patients with Metabolic Syndrome (MetS) according to the NCEP-ATP III criteria. The PC patients with higher VAI scores were subdivided into three groups according to VAI tertiles (i.e. PC patients with mild VAD, moderate VAD or severe VAD). Finally, VAD classes were compared to classical cardio- and cerebrovascular risk factors as independent predictors of coronary heart disease and/or myocardial infarction, transient ischemic attack and/or ischemic stroke.

**Results:**

Moderate and severe VADs proved to be independently associated with cardiovascular events [(OR: 5.35; 95% CI: 1.92-14.87; p = 0.001) and (OR: 7.46; 95% CI: 2.64-21.05; p < 0.001) respectively]. Mild, moderate and severe VADs were found to be independently associated with cerebrovascular events [(OR: 2.73; 95% CI: 1.12-6.65; p = 0.027), (OR: 4.20; 95% CI: 1.86-9.45; p = 0.001) and (OR: 5.10; 95% CI: 2.14-12.17; p < 0.001) respectively].

**Conclusions:**

Our study suggests that among Caucasian Sicilian subjects there are clear cut-off points of VAI able to identify a VAD strongly associated with cardiometabolic risk.

## Background

The term 'cardiometabolic risk' was coined by the American Diabetes Association [1] and the American Heart Association [2] to describe the overall risk of developing type 2 diabetes and cardiovascular diseases. Abdominal obesity, insulin resistance and 'dysfunctional' visceral fat are key components of a constellation of metabolic abnormalities, related to energy surplus (resulting from a sedentary lifestyle combined with excessive calorie consumption), characterizing 'cardiometabolic risk' [3]. To identify visceral obesity, the clinical parameter most commonly used today is Waist Circumference (WC). Nevertheless, WC alone does not help in distinguishing between subcutaneous and visceral (both omental and mesenteric) fat mass [4]. This is particularly significant given that differences in insulin-sensitivity, lipolytic activity and adipocytokines production play a fundamental role in the genesis of cardiovascular sequelae [5-7]. MRI and CT are now considered the gold standard for the quantitative evaluation of Visceral Adipose Tissue (VAT) and Subcutaneous Adipose Tissue (SAT) [8]. Since these two methods are extremely expensive and complicated to perform, they cannot be recommended in routinely clinical practice.

Furthermore, in order to predict VAT-associated cardiometabolic risk, it would be highly desirable to perform routine evaluation of "visceral adipose dysfunction" (VAD) by adipocytokine assessment. This approach, however, is also unfeasible because of the complexity of the 'adipose endocrine organ' function [9], and again for the high costs involved.

Recently our research group developed the Visceral Adiposity Index (VAI), a mathematical model that uses both anthropometric (body mass index [BMI] and WC) and functional (triglycerides [TG] and high-density lipoprotein [HDL] cholesterol) simple parameters [10]. This index, which could be considered a simple surrogate marker of VAD, showed a strong association with both the rate of peripheral glucose utilization (M value) during the Euglycemic-hyperinsulinemic Clamp and with visceral adipose tissue (VAT) measured with MRI. Furthermore, it showed a strong independent association with both cardiovascular and cerebrovascular events [10] and showed better predictive power for incident diabetes events than its individual components (WC, BMI, TG and HDL) [11]

Moreover, this index has been studied in specific populations of patients: in a specific cohort of patients with genotype 1 chronic hepatitis C, VAD identified by a higher VAI score proved to be independently associated with both steatosis and necroinflammatory activity and directly correlated with viral load [12]; in a specific cohort of women with polycystic ovary syndrome (PCOS) it has proved to be an easy and useful tool for the assessment of cardiometabolic risk associated with the oligomenorrhoic phenotype of PCOS [13].

This cross-sectional retrospective study therefore had a 2-fold objective:

1) to identify in a Caucasian Sicilian population, stratified for age, VAI cut-off points strongly associated with Metabolic Syndrome (MetS) using the receiver operating characteristic (ROC) curve analysis;

2) to examine the relationship between this surrogate and indirect VAD measure and cardio- and cerebrovascular events.

## Methods

This retrospective cross-sectional study was performed in collaboration with 10 National Health Service Primary Care Physicians (PCPs) on 13,195 individuals from the town of Alcamo, in Western Sicily (The AlkaMesy Study) as previously described [10]. Since data from the 13,195 Primary Care patients (PC patients) were recorded anonymously, no individual informed consent was needed. The study was approved by the Institutional Review Board of the Faculty of Medicine, University of Palermo, given that the identity of the participants remained anonymous during database analysis.

### Data Collection

Data have been recorded by PCPs since 1996 using the Millewin computed medical chart (v. 13.35.1054, Gruppo Dedalus Millenium srl; Florence, Italy).

For 1,764 of the 13,195 PC patients, complete information was available on the following aspects: age, sex, smoking habits, WC (cm), BMI (kg/m^2^), age of first heart and/or cerebrovascular event, diagnosis of diabetes mellitus according to the American Diabetes Association [14], presence of high blood pressure according to ESH/ESC criteria [15], dyslipidemia as defined by NCEP ATP III [16], coronary heart disease (CHD) and/or myocardial infarction (MI) and transient ischemic attack (TIA) and/or ischemic stroke (IS). This group (585 males and 1,179 females; mean age 47.80 ± 18.28, range 16-99 years) was selected for cross-sectional analysis; the number of selected PC patients was adequate to evaluate two-tailed hypotheses regarding differences in the parameters investigate between the subgroups (PC patients with VAD and without VAD) in the study greater than 0.5 standard deviations, achieving statistical power greater than 0.80 at 5% probability level (p-value).

With regard to the clinical and biochemical parameters analyzed, we used the mean value of data recorded in the last six months of follow-up. For patients who experienced cardio- and/or cerebrovascular accident, we used the mean value of data recorded in the six months before the event. Subjects who had had a cardio- and/or cerebrovascular event before 1996 (i.e. before the Mille win computed medical chart was used) were excluded because data were not available at the time of the event.

WC was measured at the midpoint between the lower rib and the iliac crest. Blood chemistry analyses were performed in accredited laboratories of the National Health Service in Alcamo.

LDL cholesterol was calculated using the Friedwald equation. Glomerular Filtration Rate (GFR) was estimated from serum creatinine using the MDRD formula and was expressed as ml/min/1.73 m^2 ^[17].

VAI score was calculated as described [10] using the following sex-specific equations, when *TG *is Triglycerides levels expressed in mmol/l and *HDL *is HDL-Cholesterol levels expressed in mmol/l:

$$Males:VAI=\left(\frac{WC}{39.68+\left(1.88\times BMI\right)}\right)\times \left(\frac{TG}{1.03}\right)\times \left(\frac{1.31}{HDL}\right)$$

$$Females:VAI=\left(\frac{WC}{36.58+\left(1.89\times BMI\right)}\right)\times \left(\frac{TG}{0.81}\right)\times \left(\frac{1.52}{HDL}\right)$$

### Statistical analysis

The Statistical Packages for Social Sciences SPSS version 17 and MedCalc version 11.3 were used for data analysis. Baseline characteristics were presented as mean ± Standard Deviation (SD) for continuous variables; rates and proportions were calculated for categorical data. Normality of distribution for quantitative data was assessed by the Kolmogorov-Smirnov test. Because HbA1c did not present normal distribution, it was therefore log-transformed. Receiver-operating characteristic (ROC) curve analyses were performed to determine appropriate cut-off points of VAI in identifying PC patients with MetS. Differences between groups in univariate analysis were detected by the unpaired Student's *t *test for continuous variables and by the χ^2^-test and Fisher's exact test (when appropriate) for categorical variables. The Anova trend analysis and the χ^2^-test for trend were used to assess means and proportions of the population characteristics across the four PC patient groups (VAD absent, Mild VAD, Moderate VAD, Severe VAD). Multiple logistic regression models were performed to explore possible determinants of cardio- and cerebrovascular events using two predictive models for dichotomic variables: "*coronary heart disease (CHD) or myocardial infarction (MI)" *and *"transient ischemic attack (TIA) or ischemic stroke (IS)"*.

Variables associated with the dependent variable on univariate analysis (probability threshold, p ≤ 0.10) were included in two multivariate regression models.

In the first model the following independent variables were included: Age and Total Cholesterol as continuous variables; gender (female = 0; male = 1), smoking (never smoker = 0; current/former smoker = 1), VAD categories (absent = 0; mild = 1; moderate = 2; severe = 3), MetS (absent = 0; present = 1), diabetes mellitus/FPG > 5.6 mmol/l (absent = 0; present = 1), high blood pressure (absent = 0; present = 1) as categorical (dichotomic and ordinal) variables. In the second model the following independent variables were included: age and GFR as continuous variables; gender (female = 0; male = 1), VAD categories (absent = 0; mild = 1; moderate = 2; severe = 3), MetS (absent = 0; present = 1), diabetes mellitus/FPG > 5.6 mmol/l (absent = 0; present = 1), high blood pressure (absent = 0; present = 1) as categorical (dichotomic and ordinal) variables. To avoid effects of multicollinearity with VAD categories the variables High Tryglicerides, Low Hdl Cholesterol, Increased WC and BMI were not included in either regression model. A *P *value of <0.05 was considered statistically significant.

## Results

**Figure 1:**
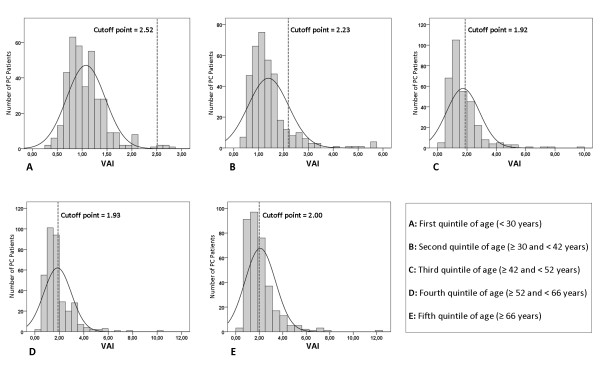
**Age-stratified cut-off points of VAI for identification of PC patients with Metabolic Syndrome according to the NCEP-ATP III criteria**.

**Figure 2:**
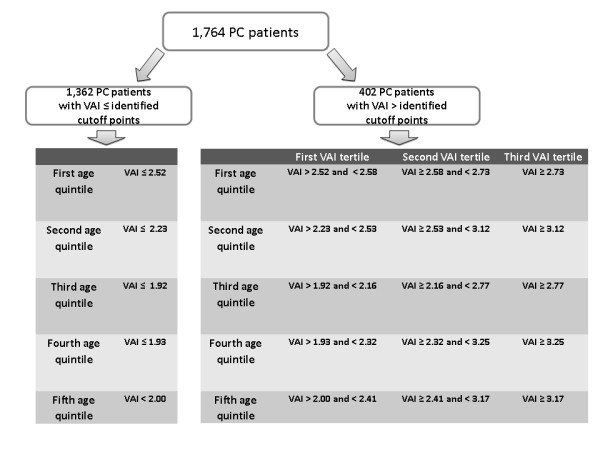
**Subdivision of 1,764 PC patients according to identified cut-off points of VAI and age quintiles**.

The 1,764 PC patients (585 males and 1,179 females; mean age 47.80 ± 18.28, range 16-99 years; mean BMI 24.27 ± 4.03; mean WC 84.70 ± 11.92) were subdivided into age quintiles in order to identify optimal age-stratified cut-off points of VAI identifying the presence of MetS. Optimal VAI cut-off points were: 2.52 (age < 30 years), 2.23 (age ≥ 30 and < 42 years), 1.92 (age ≥ 42 and < 52 years), 1.93 (age ≥ 52 and < 66 years) and 2.00 (age ≥ 66 years) (Table 1, Figure 1). The PC patients who had a VAI score over the age-stratified cut-off points were 402 (22.78%). This group was divided into VAI tertiles as shown in Figure 2. The 1,362 PC patients with VAI less than or equal to age-stratified cut-off points were defined "*with VAD absent"*; in the remaining 402 subjects, the first VAI tertile of PC patients was defined "*with mild VAD"*, the second tertile "*with moderate VAD" *and the third tertile "*with severe VAD" *(Table 2).

**Table 1:** Optimal cut-off points of VAI (stratifying PC Patients for age quintiles) to detect subjects with Metabolic Syndrome (ATP III criteria).

	Cutoff Point	Sens. (%)	Spec. (%)	Area under ROC curve	SE	95% CI	p
**First age quintile** **Age (< 30 years)**	2.52	100	99.45	0.997	0.003	0.98 - 1.00	< 0.001
**Second age quintile** **Age (≥ 30 and < 42 years)**	2.23	84.62	92.39	0.898	0.061	0.86 - 0.92	< 0.001
**Third age quintile** **Age (≥ 42 and < 52 years)**	1.92	90.48	72.55	0.852	0.037	0.80 - 0.88	< 0.001
**Fourth age quintile** **Age (≥ 52 and < 66 years)**	1.93	77.22	82.29	0.840	0.028	0.79 - 0.87	< 0.001
**Fifth age quintile** **Age (≥ 66 years)**	2.00	68.5	76.0	0.783	0.025	0.73 - 0.82	< 0.001

Sens. (sensitivity); Spec. (Specificity); SE (Standard Error). CI (Confidence Interval)

**Table 2:** Features of 1,764 PC patients grouped according to VAD classes.

	PC patients with VAD absent No 1362	PC patients with Mild VAD No 126	PC patients with Moderate VAD No 141	PC patients with Severe VAD No 135	
					*p*^*§*^
**Females/Males**	903/459	84/42	102/39	90/45	*0.427*
	*Mean ± SD*	*Mean ± SD*	*Mean ± SD*	*Mean ± SD*	
**Age (years)**	44.46 ± 17.80	58.81 ± 14.56	59.22 ± 15.32	59.28 ± 15.38	*< 0.001*
**BMI (Kg/m**^**2**^**)**	23.71 ± 3.62	26.14 ± 4.68	25.92 ± 4.86	26.53 ± 4.61	*< 0.001*
**WC (cm)**	82.73 ± 10.76	90.94 ± 12.51	90.65 ± 13.89	92.52 ± 13.09	*< 0.001*
	*No (%)*	*No (%)*	*No (%)*	*No (%)*	
Metabolic Syndrome*	62 (4.6)	40 (31.7)	52 (36.9)	88 (65.2)	*< 0.001*
**Diabetes or fasting glucose ≥ 5.6 mmol/l**	184 (13.5)	43 (34.1)	41 (29.1)	52 (38.5)	*< 0.001*
**Only diabetes**	64 (4.7)	22 (17.5)	22 (15.6)	29 (21.5)	*< 0.001*
**High Blood pressure**	262 (19.2)	55 (43.7)	62 (44)	73 (54.1)	*< 0.001*
**High Triglycerides**	31 (2.3)	41 (32.5)	80 (56.7)	123 (91.1)	*< 0.001*
**Low HDL Cholesterol**	215 (15.8)	50 (39.7)	81 (57.4)	92 (68.1)	*< 0.001*
**Increased WC**	165 (12.1)	38 (30.29)	49 (34.8)	54 (40.0)	*< 0.001*
Classes of obesity**					
**Underweight**	16 (1.2)	1 (0.8)	1 (0.7)	-	*0.178*
**Normal weight**	1052 (77.2)	68 (54)	75 (53.2)	67 (49.6)	*< 0.001*
**Overweight**	209 (15.3)	39 (31)	42 (29.8)	45 (33.3)	*< 0.001*
**Obese class I**	65 (4.8)	10 (7.9)	16 (11.3)	18 (13.3)	*< 0.001*
**Obese class II**	19 (1.4)	7 (5.6)	6 (4.3)	2 (1.5)	*0.082*
**Obese class III**	1 (0.1)	1 (0.8)	1 (0.7)	3 (2.2)	*< 0.001*
					
**CHD or MI**	13 (1)	7 (5.6)	10 (7.1)	14 (10.4)	*< 0.001*
**TIA or IS**	21 (1.5)	9 (7.1)	14 (9.9)	15 (11.1)	*< 0.001*
	*Mean ± SD*	*Mean ± SD*	*Mean ± SD*	*Mean ± SD*	
**Fasting glucose (mmol/l)**	5.11 ± 1.26	5.71 ± 1.66	5.59 ± 1.77	5.80 ± 2.37	*< 0.001*
**AST (UI/l)**	21.46 ± 19.45	24.03 ± 17.85	20.11 ± 6.39	25.48 ± 22.39	*0.104*
**ALT (UI/l)**	20.99 ± 11.69	24.45 ± 17.33	20.32 ± 7.80	25.52 ± 19.24	*0.002*
**GFR (ml/min/1.73 m**^**2**^)***	73.11 ± 25.78	65.14 ± 25.79	60.19 ± 33.65	64.53 ± 32.29	*< 0.001*
**Total Cholesterol (mmol/l)**	4.88 ± 0.75	5.12 ± 0.94	5.35 ± 0.99	5.51 ± 0.95	*< 0.001*
**HDL cholesterol (mmol/l)**	1.41 ± 0.24	1.25 ± 0.22	1.18 ± 0.22	1.10 ± 0.23	*< 0.001*
**Triglycerides (mmol/l)**	0.99 ± 0.31	1.56 ± 0.32	1.78 ± 0.38	2.72 ± 0.97	*< 0.001*
LDL Cholesterol (mmol/l)****	3.01 ± 0.69	3.15 ± 0.86	3.35 ± 0.91	3.16 ± 0.97	*< 0.001*
**HbA1c (%) Only in subgroup of diabetic patients**	6.45 ± 1.40	7.04 ± 1.45	6.79 ± 1.18	7.48 ± 1.50	*0.361*

* According to Adult Treatment Panel (ATP) III criteria; ** WHO classification; *** Calculated by *Modification of Diet in Renal Disease *(MDRD) equation;**** Calculated by Friedewald formula. ^§ ^p value for trend

VAD (visceral adipose dysfunction), BMI (body mass index), WC (waist circumference), HDL (high-density lipoprotein), LDL (low-density lipoprotein), CHD (coronary heart disease), MI (myocardial infarction), TIA (transiet ischemic attack), IS (ischemic stroke), AST (aspartate transaminase), ALT (alanine transaminase)

A significant increasing trend between the four groups of PC patients (PC patients with absent VAD, mild VAD, moderate VAD and severe VAD) was observed for age (p < 0.001), BMI (p < 0.001), WC (p < 0.001), fasting glucose (p < 0.001), ALT (p = 0.002), total cholesterol (p < 0.001) and LDL cholesterol (p < 0.001). The prevalence of patients with MetS, diabetes, high blood pressure, low HDL cholesterol, high triglycerides, CHD and/or MI, TIA and/or IS, increased significantly across the four groups (p < 0.001 for all variables). A significant reducing trend was seen for GFR (p < 0.001) (Table 2). To validate VAD classes as potential indicators of visceral fat dysfunction associated with cardiovascular (CHD and/or MI) and cerebrovascular events (TIA and/or ischemic stroke), data from the 1,764 PC patients were analyzed using two binary logistic regression models (Table 3). Among all independent variables examined *moderate VAD *(OR = 5.35; 95% CI: 1.92 - 14.87; p = 0.001), *severe VAD *(OR = 7.46; 95% CI: 2.64 - 21.05; p < 0.001), age at the time of event (OR = 1.06; 95% CI: 1.03 - 1.08; p < 0.001), smoking (OR = 3.34; 95% CI: 1.54 - 7.24; p = 0.002) and male gender (OR = 2.95; 95% CI: 1.33 - 6.54; p = 0.007) were independently correlated with cardiovascular events (CHD and/or MI). With regard to cerebrovascular events (TIA and/or IS), *mild VAD *(OR = 2.73; 95% CI: 1.12 - 6.65; p = 0.027), *moderate VAD *(OR = 4.20; 95% CI: 1.86 - 9.45; p = 0.001), *severe VAD *(OR = 5.10; 95% CI: 2.14 - 12.17; p < 0.001), age at time of event (OR = 1.07; 95% CI: 1.05 - 1.10; p < 0.001), male gender (OR = 2.47; 95% CI: 1.32 - 4.63; p = 0.005) showed an independent association; a weak inverse independent association was found with GFR (OR = 0.98; 95% CI: 0.97 - 1.00; p = 0.047) (Table 3).

**Table 3:** Univariate and multivariate analysis (logistic regression models) of risk factors associated with "Coronary heart disease (CHD) and/or myocardial infarction (MI)" and "Transient ischemic attack (TIA) and/or Ischemic Stroke (IS)" in 1,764 PC patients.

Coronary Heart Disease (CHD) and/or Myocardial Infarction (MI)					
	**PCP** **Without CHD/MI** No 1720	**PCP** **With CHD/MI** No 44	Univariate analysis	Multivariate Analysis	
	*Mean ± SD*	*Mean ± SD*	*p*	*p*	*OR (IC 95%)*
**Age (years)**	47.15 ± 17.98	67.64 ± 10.90	< 0.001	< 0.001	1.06 (1.03 - 1.08)
**BMI**	24.21 ± 4.00	26.70 ± 4.46	< 0.001	-	-
**Total Cholesterol**	4.97 ± 0.83	5.33 ± 0.87	0.005	0.774	1.05 (0.73 - 1.52)
**GFR (ml/min/1.73 m**^**2**^**)**	71 ± 27.13	65.06 ± 34.55	0.155	-	-
	*No (%)*	*No (%)*			
**Gender Females Males**	1160 (67.4) 560 (32.6)	19 (43.2) 25 (56.8)	0.001	0.007	2.95 (1.33 - 6.54)
**Current or former smoker**	520 (32.1)	24 (61.5)	< 0.001	0.002	3.34 (1.54 - 7.24)
**VAD absent Mild VAD Moderate VAD Severe VAD**	1349 (78.4) 119 (6.9) 131 (7.6) 121 (7)	13 (29.5) 7 (15.9) 10 (22.7) 14 (31.8)	< 0.001	0.158 0.001 <0.001	2.37 (0.71 - 7.88) 5.35 (1.92 - 14.87) 7.46 (2.64 - 21.05)
**Metabolic Syndrome**	220 (12.8)	22 (50)	< 0.001	0.581	1.36 (0.45 - 4.11)
**Diabetes or fasting glucose ≥ 5.6 mmol/l**	303 (17.6)	17 (38.6)	< 0.001	0.403	0.678 (0.27 - 1.68)
**High Blood pressure**	424 (24.7)	28 (63.6)	< 0.001	0.278	1.61 (0.68 - 3.83)
**High Triglycerides**	259 (15.1)	16 (36.4)	< 0.001	-	-
**Low HDL Cholesterol**	405 (23.5)	33 (75)	< 0.001	-	-
**Increased WC**	290 (16.9)	16 (36.4)	0.001	-	-
**Transient ischemic attack (TIA) and/or Ischemic Stroke (IS)**					
	**PCP** **Without TIA/IS** No 1705	**PCP** **With TIA/IS** No 59	Univariate analysis	Multivariate Analysis	
	*Mean ± SD*	*Mean ± SD*	*p*	*p*	*OR (IC 95%)*
**Age (years)**	46.84 ± 17.73	71.91 ± 11.11	< 0.001	< 0.001	1.07 (1.05 - 1.10)
**BMI**	24.24 ± 4.02	25.25 ± 4.32	0.058	-	-
**Total Cholesterol**	4.98 ± 0.83	5.02 ± 0.81	0.762	-	-
**GFR (ml/min/1.73 m**^**2**^**)**	71.38 ± 27.20	55.75 ± 27.43	< 0.001	0.047	0.98 (0.97 - 1.00)
	*No (%)*	*No (%)*			
**Gender Females Males**	1146 (67.2) 559 (32.8)	33 (55.9) 26 (44.1)	0.070	0.005	2.47 (1.32 - 4.63)
**Current or former smoker**	525 (32.7)	19 (36.5)	0.561	-	-
**VAD absent Mild VAD Moderate VAD Severe VAD**	1341 (78.7) 117 (6.) 127 (7.4) 120 (7)	21 (35.6) 9 (15.3) 14 (23.7) 15 (25.4)	< 0.001	0.027 0.001 < 0.001	2.73 (1.12 - 6.65) 4.20 (1.86 - 9.45) 5.10 (2.14 - 12.17)
**Metabolic Syndrome**	215 (12.6)	27 (45.8)	< 0.001	0.634	0.813 (0.34 - 1.90)
**Diabetes or fasting glucose ≥ 5.6 mmol/l**	295 (17.3)	25 (42.4)	< 0.001	0.584	1.21 (0.60 - 2.44)
**High Blood pressure**	411 (24.1)	41 (69.5)	< 0.001	0.078	1.89 (0.93 - 3.83)
**High Triglycerides**	249 (14.6)	26 (44.1)	< 0.001	-	-
**Low HDL Cholesterol**	401 (23.5)	37 (62.7)	< 0.001	-	-
**Increased WC**	289 (17)	17 (28.8)	0.018	-	-

**Univariate analysis**: qualitative variables were analyzed through χ^2 ^test or Fisher exact Test; quantitative variables were analyzed through Student's *t *Test. Independent variables showing p value ≤ 0.10 in univariate analysis were entered in multivariate analysis. To avoid effects of multicollinearity with VAD categories the variables *High Triglycerides*, *Low HDL Cholesterol*, *Increased WC *and *BMI *were not included in either regression model.

PCP (primary care patients), VAD (visceral adipose dysfunction), GFR (Glomerular filtration rate) BMI (body mass index), WC (waist circumference), HDL (high-density lipoprotein), CHD (coronary heart disease), MI (myocardial infarction), TIA (transiet ischemic attack), IS (ischemic stroke).

## Discussion

In our previous study we suggested that a high VAI score is associated with cardiovascular and cerebrovascular events, well identifying patients with cardiometabolic risk [10]. However, given the lack of a prospective longitudinal study, it was not possible to define irrefutably a high VAI score as a cardiovascular risk factor. In fact, the cross-sectional nature of the study has not made it possible to find causal inferences regarding the relationship between VAI and cardio- and cerebrovascular events.

In this study we identified in a Caucasian Sicilian population the age-stratified cut-off points of VAI which proved to be strongly associated with MetS. The appropriate cut-off points of VAI for detecting MetS were 2.52 for PC patients under 30 years, 2.23 for those aged between 30 and 42 years, 1.92 between 42 and 52 years, 1.93 between 52 and 66 years and 2.00 for PC patients over 66 years.

PC patients with VAI scores greater than these cut-off points were arbitrarily defined as having mild, moderate or severe *visceral adipose dysfunction *(VAD).

Since visceral fat is universally claimed to be more strongly associated with cardiometabolic risk than subcutaneous fat, we believe that emphasis should be given to the new model represented by VAD, since it is widely reported that visceral fat is more strongly associated with cardiometabolic risk compared with subcutaneous fat [18,19] and that VAT remains confirmed as an important correlate of metabolic risk factors after accounting for BMI [18,20]. Furthermore, over the last decade there has been increasing evidence regarding the endocrine function of adipose tissue: changes in the secretory function of adipocytes and macrophages, together with chronic, low-grade inflammation, are associated with insulin resistance, dyslipidemia, diabetes and/or vascular disease, contributing to the clinical effects of obesity [21-23].

PC patients with VAD ranging from mild to severe, showed a significant trend of increased prevalence of all components of the MetS and of cardio- and cerebrovascular events. A significant increased trend for age, fasting glucose, ALT, total cholesterol, TG and HDL was also observed.

Moderate and severe VAD proved to be independently associated with cardiovascular (CHD and/or MI) events, together with smoking, male gender and age at the time of events, when compared with other conventional dichotomic risk factors, such as diabetes mellitus/FPG > 5.6 mmol/l, high blood pressure, total cholesterol and MetS. Furthermore, only mild, moderate and severe VAD together with male gender and age at the time of events proved to be independently associated with cerebrovascular (TIA and/or IS) events.

Surprisingly, VAD was independently associated with cardio- and cerebrovascular events, but not with MetS diagnosed according to the ATP III criteria. A possible explanation might lie in the fact that the 5 variables proposed in the ATP III criteria are not used as quantitative continuous variables, but rather as dichotomous variables. Thus, such screening tools might prove to be less efficient for the optimal assignment of a patient with cardiometabolic risk related to his/her visceral obesity. Indeed, the 'presence' or the 'absence' of an abnormality may be too crude to identify the individual risk profile. Lastly, with current Mets diagnosis criteria, tools are considered as a homogeneous entity, which is very unlikely, especially as regards diabetes and high blood pressure [24,25].

For VAI, which represents a global calculator of non-glycemic and non-hemodynamic components of the MetS, while on one hand the variables are treated as continuous variables, on the other two important aspects are taken into consideration, i.e. BMI and gender. In fact, women have, on average, more subcutaneous fat and less visceral fat than men [26].

Although VAI cannot be claimed *per se *as a diagnostic tool for cardiovascular and cerebrovascular events, since it includes physical (BMI and WC) and metabolic (TG and HDL) parameters, it may, however, indirectly reflect other non-classical risk factors, i.e. altered production of adipocytokines, increased lipolytic activity and plasma-free fatty acids. In fact, visceral obesity and "High-Triglyceride/Low-HDL-Cholesterol Dyslipidemia" were proposed by Unger et al. [27], who suggested that a state of relative hypoleptinemia can be observed in subjects with visceral obesity compared with generalized obesity. This condition, which we ourselves consider useful to define *visceral fat dysfunction*, when associated with physiological age-linked leptin resistance, leads to pancreatic lipotoxicity with subsequent beta-cell apoptosis and diabetes onset, muscle insulin resistance, liver insulin resistance and NAFLD, lipotoxic cardiomyopathy and generalized endothelial dysfunction [27].

## Conclusion

In conclusion, given the simplicity of WC and BMI measurement and TG and HDL assessment, and the identification of reference cut-off points in a Caucasian population, we suggest that VAI would be an easy tool for the assessment of VAD, and might be useful in daily clinical practice and in population studies for the assessment of cardiometabolic risk associated with visceral obesity.

## Competing interests

The authors declare that they have no competing interests.

## Authors' contributions

All authors contributed to the intellectual development of this work, and approved the final manuscript. MCA designed the study, performed the statistical analysis of data, searched the literature and wrote the draft paper; MP searched the literature; CG and AG coordinated the study, searched the literature and provided critical corrections to the manuscript.
